# Herbicide Widespread: The Effects of Pethoxamid on Nonalcoholic Fatty Liver Steatosis *In Vitro*

**DOI:** 10.1155/2020/7915795

**Published:** 2020-09-04

**Authors:** Anna Virginia Adriana Pirozzi, Antonietta Stellavato, Chiara Schiraldi, Mariateresa Giuliano

**Affiliations:** Department of Experimental Medicine, Section of Biotechnology, Medical Histology and Molecular Biology, University of Campania “L. Vanvitelli”, Via De Crecchio 7, 80131 Naples, Italy

## Abstract

Pethoxamid is a widespread herbicidal product, presenting itself as an extremely flexible active substance and with a high potential for use as an herbicide for preemergence. The emergence of multiple resistance in crops has been addressed using combinations of preemergence and postemergence herbicides in the same seeding-harvest cycle. A winning combination of pethoxamid and glyphosate mainly affected the acidobacteria population. Glyphosate scientific literature has demonstrated an observational link between herbicide exposure and liver disease in human subjects. Identifying and ranking the risk to the public that pethoxamid could exert on target organs has not been evaluated so far. Due to similarities to glyphosate, we did look at the effect of pethoxamid on impaired liver cells HepG2, using a nonalcoholic fatty liver disease (NAFLD) cell model *in vitro*. Pethoxamid was cytotoxic starting at 1 ppm. Fatty acid accumulation (FA) was enhanced while low doses of pethoxamid slightly decreased LDH protein expression compared to FA-treated HepG2. The same trend was observed for cytochrome c. Based on our data, we can argue that NAFLD hepatic cells react to pethoxamid trying detoxifying strategies, ready to undergo cell death to avoid further degeneration. Downregulation of cytochrome can lead to the hypothesis that pethoxamid should not induce herbicide resistance.

## 1. Introduction

Intensive farming requires the use of large quantities of pesticides that reach the general population through several ways. Dissipation studies have focused on the soil microbial structure, on the adsorption, and on the residues generated by different environmental factors [1].

Linked to chemical research, the chemical risk assessment has been improved to limit human and environmental safety decline.

Pethoxamid (2-chloro-N-(2-ethoxyethyl)-N-(2-methyl-1-phenylprop-1-enyl) acetamide) is a substance that was included in Annex I to Council Directive 91/414/EEC concerning the placing of plant protection products on the market, by Commission Directive 2006/41/EC4. Pethoxamid is deemed to have been approved under Regulation (EC) No 1107/2009 and is listed in Part A of the Annex to Commission Implementing Regulation (EU) No 540/20115 [2].

Its molecular action mechanism consists in the block of cell cycle that prevents the growth of unwanted plants (various grasses and broadleaf weeds) and is intended for use before or just after their germination in corn, rice, and soy.

Pethoxamid presents itself as an extremely flexible active substance and with a high potential for use as an herbicide for preemergence, in relation to the many possible fields of use. Pethoxamid is very effective on the main grass weeds and numerous dicotyledons.

Pethoxamid does not possess carcinogenic, mutagenic, and teratogenic activities. There are no specific risks for the operator once all the precautions normally required and indicated on the label are observed. Pethoxamid has a half-life of only 22–24 days. It is a perfect balance between persistence of action and the absence of risks for any succession of crops. Moreover, such a residence time in soil limits the possibility to spread into the environment and into surface waters or groundwater.

The emergence of multiple resistance in crops have been addressed using combinations of preemergence and postemergence herbicides in the same seeding-harvest cycle. The combination of pethoxamid and glyphosate mainly affected the acidobacteria population [3]. The use of herbicide combinations may exert synergistic effects not seen in time-separated administration. These mixtures need a significant knowledge of the relative impact on environmental and human health.

Glyphosate's rich scientific literature has demonstrated an observational link between herbicide exposure and liver disease in human subjects [4]. Alterations in metabolomics and proteomics are present in nonalcoholic fatty liver disease (NAFLD) and its progression to steatohepatitis (serious fatty liver disease) [5].

Similarities between glyphosate and pethoxamid may suggest to look and liver impairment [6–8].

Pethoxamid is mainly absorbed by oral route (>80), concentrated in tissues responsible for metabolism and excretion (gastrointestinal tract, liver, and kidneys), with indication of a possible binding to red blood cells. Mainly eliminated via bile and urine, pethoxamid did not show any bioaccumulation potential and was extensively metabolized to cysteine conjugates, sulfoxides, and sulfones. According to EFSA, the available data could not conclude on pethoxamid toxicity leading to a data gap [8].

Identifying and ranking the risk to the public that pethoxamid could exert on target organs has not been evaluated so far. Particularly, no data are available on human liver toxicity focusing on susceptible unhealthy liver.

NAFLD is predicted to be the next major global epidemic, currently affecting 25% of the US and Europe populations [5, 9–11]. Risk factors include being overweight or obese, having diabetes, high cholesterol, or high triglycerides in the blood. Rapid weight loss and poor eating habits also may lead to NAFLD. Still occurrence of NAFLD has been observed in the absence of the mentioned risk factors [12].

*In vitro* models of organ-derived cell cultures are a cutting-edge tool of biomedical research and proved essential for toxicological screening [13–15].

The well-established hepatocarcinoma cell line HepG2 has been used as a key to study NAFLD pathophysiology [11, 15, 16].

The aim of this work was to assess selected biochemical markers of alert to pethoxamid by impaired liver cells using an hepatocarcinoma (HepG2)-based model of NAFLD.

## 2. Material and Methods

### 2.1. Cell Culture and Treatments

The hepatocarcinoma cell line HepG2 and cell culture materials were obtained from Sigma-Aldrich (Milan, Italy), unless otherwise stated. Eagle's Minimum Essential Medium (EMEM) with 10% fetal bovine serum (FBS), 100 U/mL penicillin, 2 mM glutamine, and 1% nonessential amino acids was used for cell culture. Tissue culture plates (BD Biosciences-Falcon, San Jose, CA, USA) were incubated with a humidified atmosphere (95% air/5% CO_2_ v/v) at 37°C. Steatosis was induced by stimulating the hepatocytes with 6 mM of a 1 : 1 v/v mixture of oleic (18 : 1) and linoleic (18 : 2) fatty acids (FAs) for 24 hours. Cells were exposed for an additional 24 hours to pethoxamid (solubilized in 100% DMSO diluted to 0.5% in serum-free DMEM) at 0.1, 1, 10, 100, or 500 ppm.

### 2.2. MTT Assay

The reduction of the tetrazolium dye 3-(4, 5-dimethylthiazol-2-yl)-5-(3 carboxymethoxyphenyl)-2-(4-sulfophenyl)-2H-tetrazolium (MTT) was used for cytotoxicity assessment. Briefly, mitochondrial dehydrogenates of living cells reduce the tetrazolium ring, yielding a blue formazan product that can be measured spectrophotometrically. The optical densities obtained are directly proportional to the number of living cells. The cytotoxic effect is reported as percentage of living cells after exposure to pethoxamid with respect to vehicle-treated cells [17].

### 2.3. Oil Red O Staining

FAs-treated HepG2 cells were exposed to pethoxamid as described above. Cold phosphate buffered saline was used to wash the cells before fixing in 4% v/v paraformaldehyde for 30 min and staining with oil red O (0.5% v/v). Images of cells were captured using an optic microscope, lipid droplets were extracted with isopropanol (60% v/v) and quantified spectrophotometrically at 510 nm [16].

### 2.4. Microscopy

Pethoxamid-treated cells were photographed using an optic microscope (Niko Eclipse TS 100), 10*X* magnification, and compared to untreated cells before performing MTT assay. For oil red O staining, cells pictures were obtained by an optic microscope (Niko Eclipse TS 100) before lipid droplets extraction and spectrophotometric quantification.

### 2.5. Western Blotting

RIPA lysis buffer was used for protein extraction while concentration was determined with Bio-Rad protein assay reagent (Bio-Rad Laboratories, Milan, Italy). Equal amounts of protein (30 *µ*g) were separated on SDS-PAGE gels and blotted onto nitrocellulose filters. Specific antibodies (Santa Cruz Biotechnology, CA, USA) against lactate dehydrogenase (LDH; rabbit polyclonal IgG, H-160; 1 : 1000 v/v), cytochrome c (Cyt C; rabbit polyclonal IgG, H-1049; 1 : 500 v/v) and actin (goat polyclonal IgG, I-19; 1 : 1000 v/v) stained the filters at room temperature for 2 h. Membranes were washed three times for 10 min and incubated with a 1 : 10000 dilution of horseradish peroxidase-conjugated anti-rabbit or anti-goat antibodies for 1 h. Blots were developed using the ECL system (Amersham Biosciences, Amersham, UK) according to the manufacturer's protocol [15].

### 2.6. Statistical Analysis (*t*-Test)

All of the experimental results were expressed as mean ± standard deviation (SD) of at least three independent determinations for each experiment. Student's *t*-tests were run to search for statistical significance. *P* values ^*∗*^ < 0.05 and ^*∗∗*^ < 0.01 were considered to be significant.

## 3. Results

### 3.1. HepG2 Cell Viability

**Figure 1:**
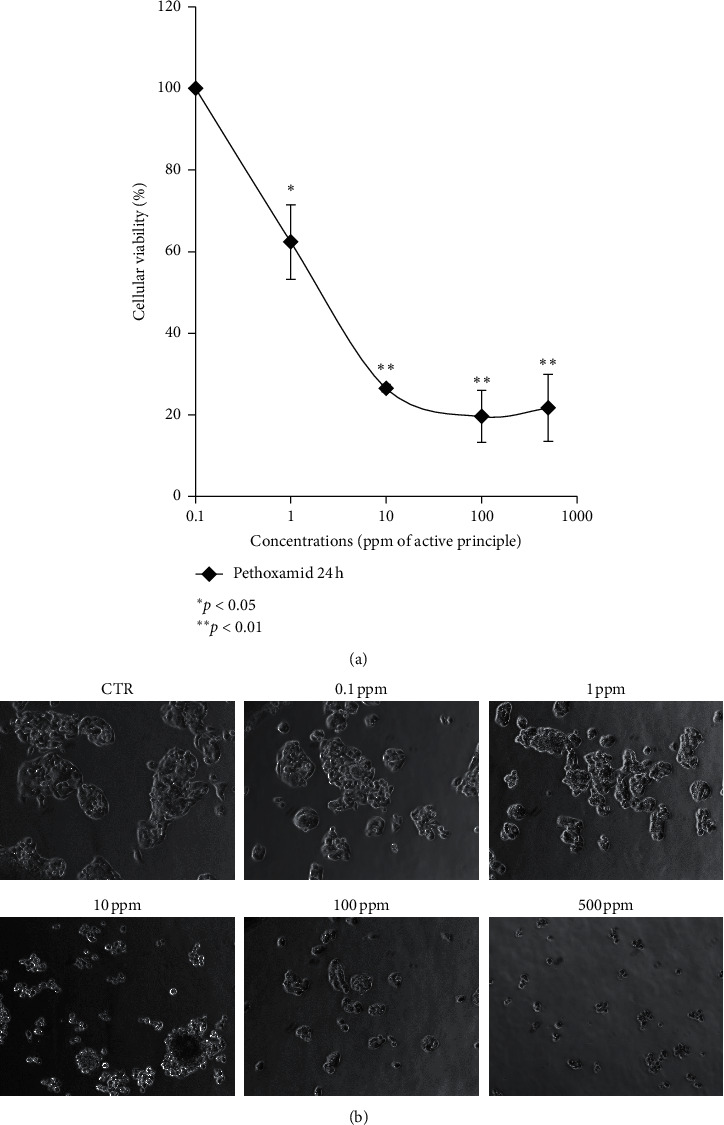
(a) Dose-dependent cytotoxicity of pethoxamid. Cell viability was significantly affected starting at 10 ppm in 24 h. Values are means ± SD from three separate experiments. (b) Representative HepG2 pictures panel in the presence of pethoxamid at different concentrations. ^*∗*^*P* < 0.05 and ^*∗∗*^*P* < 0.01*t*-test analyses were performed to compare the significance of each treatment with respect to CTR.

Cell viability was evaluated using MTT assay after 24 h of exposure to pethoxamid. Liver-impaired cells showed both significant low-dose toxicity and high-dose toxicity when treated with the tested range of pethoxamid (Figure 1(a)). Interestingly, increasing the load of pethoxamid (from 10 to 500 ppm) ended in a flat trend. Microscopy images showed morphological alterations due to pethoxamid exposure (Figure 1(b)).

### 3.2. Intracellular Lipid Accumulation

**Figure 2:**
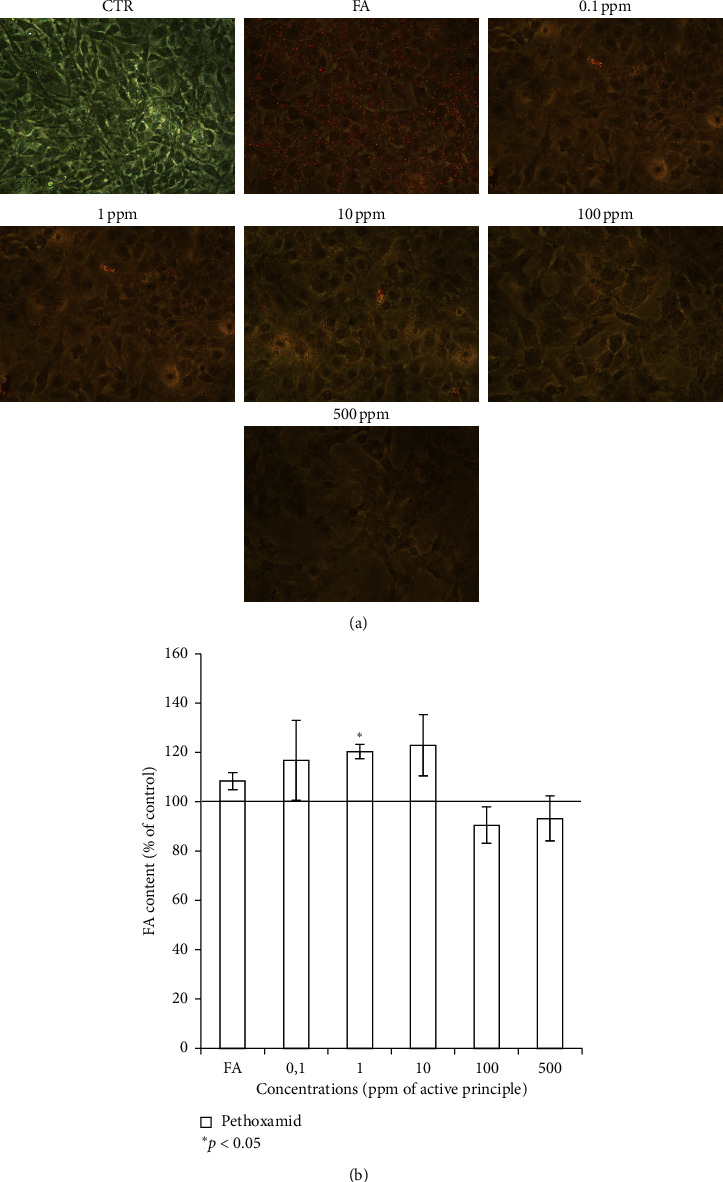
Oil red staining showed an increased steatosis at low-dose exposure. (a) Oil red O staining pictures for HepG2 cells in presence of fatty acids (FA) 6 mM, and pethoxamid-treated cells at different concentrations (0.1–500 ppm). (b) Spectophotometric quantification of lipid amount percentage with respect to the control of steatotic cells (FA 6 mM). ^*∗*^*P* < 0.05*t*-test analyses were performed to compare the significance of each treatment with respect to CTR. Values are means ± SD from three separate experiments.

The impact of pethoxamid on liver steatosis was evidenced by the intracellular lipid accumulation using oil red O staining. Pethoxamid enhanced the intracellular storage of lipid droplets, respect to FAs treatment alone at low-dose exposure (Figure 2(a)). Spectrophotometric quantification of lipid amount in high-dose treated cells seemed to protect from FAs but these data need to be associated to cell viability (Figure 2(b)).

### 3.3. Lactate Dehydrogenase and Cytochrome c Evaluation

**Figure 3:**
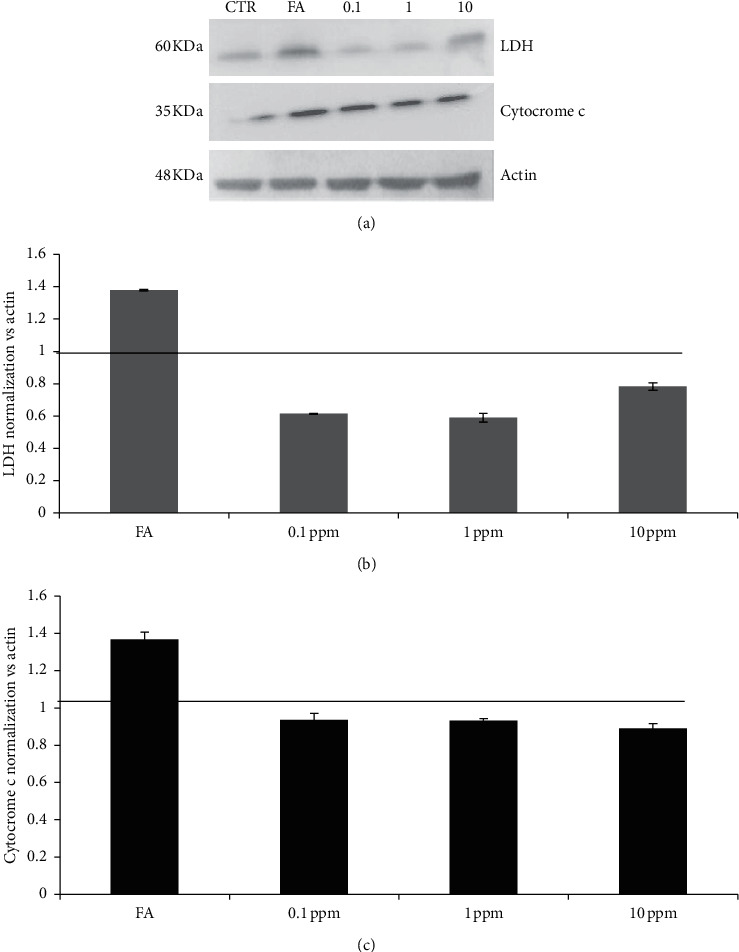
Western blot for LDH and cytochrome c. Densitometry plot (a) showing an inverse dose-dependent reduction of LDH levels in pethoxamid-treated cells. Densitometry plot (b, c) demonstrating LDH and cytochrome c reduction. Actin was used to normalize the results. Values are means ± SD from three separate experiments.

The production of lactate dehydrogenase (LDH) in hepatocytes was used as cell damage marker. Low doses of pethoxamid slightly reversed LDH protein expression compared to FA-treated HepG2 (Figure 3). The same trend was observed for cytochrome c. It was upregulated by FA treatment and the exposure to pethoxamid reduced the expression (Figure 3). Actin was used to determine loading and normalization was ran to highlight expression level. The effects of high doses of pethoxamid could not be analyzed due to massive cell death.

## 4. Discussion

Herbicides, also commonly known as weed killers, are chemical substances used to control unwanted plants [18]. Pethoxamid is a chloroacetamide, and it is a herbicidally active substance.

Herbicide overuse has resulted in the widespread occurrence of herbicide-resistant weed populations.

The excessive use of herbicides, e.g., for barnyard grass and red rice, has led to herbicide resistance making contrast to growth of weed species extremely hard. Alternative herbicide sites of action (SOAs) have been developed to be incorporated into rice, whenever possible. Pethoxamid is a valid alternative for use in rice to allow for increased rotation of herbicide SOAs to combat herbicide-resistant and difficult-to-control weeds [19].

According to EC restrictions on pethoxamid, the future use is subjected to confirmatory information on the endocrine disrupting potential. These data should be submitted by 10 November 2020 at the latest [2]. So, additional studies are required on toxicity to be submitted to the member states in order to ensure authorizations for use under certain conditions.

Accumulation data have identified liver as the target organ, with no demonstrated toxicity, to our knowledge, still not considering the increasingly frequent hepatic deficit known as NAFLD.

Basically NAFLD is linked to steatosis, with an accumulation of lipid droplets (LDs) within hepatocytes. This intrahepatocellular storage of lipids derives from elevated delivery or synthesis of FAs, reduced secretion or oxidation of FAs, or both [11].

Primary human hepatocytes are hardly available, so the present study employed a NAFLD cellular model *in vitro* based on HepG2 cell line to examine the cytotoxicity of the widely used herbicide, pethoxamid [11, 20].

In rats treated with chloroacetamide, lipid peroxidation can be significantly increased without causing permanent damage to the hepatocytes [21].

High doses of pethoxamid affected the liver and thyroid, as observed in short-term toxicity studies with rats and mice [8]. EFSA report 2017 recommended further consumer risk assessment studies.

We investigated the effects of pethoxamid on the liver, using a hepatocarcinoma-based model of NAFLD, *in vitro*. We aimed to explore the effects on hepatic cells thinking to the prolonged exposure not limited to agricultural workers, but in all those that come into contact with it or its residues, through the food chain.

Madsedn et al. reported the occurrence of an occupational allergic contact dermatitis by pethoxamid [22].

Pethoxamid is related to chloroacetamide, a known sensitizer. Incubation of chloroacetamide-based herbicides with liver microsomes showed significant differences between human and rat metabolic capacities. Moreover, human liver microsomes were able to discriminate among parent molecules [23].

The results of pethoxamid toxicity tests on the common carp *Cyprinus carpio* showed how xenobiotics could act as inducers or inhibitors of detoxification systems, including cytochromes [24].

To the best of our knowledge, this is the first time that data on pethoxamid low doses exposure on human cells, *in vitro*, are discussed to fill, at least in part, the existing gaps.

Cytoxicity data showed a strong impact on cell viability, being dramatic starting at 10 ppm. Worth of notice, the flat trend was observed when using 10–500 ppm. There may be a small percent of cells resistant to pethoxamid. High doses of pethoxamid affected the fatty acid content in a dose-dependent manner. In fact, oil red staining demonstrated that lipid accumulation was increased in the presence of low doses of pethoxamid showing a decrease at high-dose (100, 500 ppm). These results need to take into account, the strong toxicity in terms of growth-curve, so it is not worthy to speculate on but stimulated us to run western blot assays using only 0.1, 1, and 10 ppm.

Then, western blots on metabolic impairment of HepG2 refer only to low-dose exposures.

Our data imply that low-dose exposure of liver cells is not associated with marked alterations of cytochrome c and being protective in the terms of LDH level. LDH is an oxidoreductase enzyme found in nearly all living cells, related to anaerobic respiration. Hypoxic conditions increased the LDH level in various cell lines [25]. This is not the case in our experimental setting, with HepG2 under constant air exposure. Our data may, most likely, match with *in vivo* data, where elevated serum levels of LDH in patients with hepatitis have merely been regarded as a result of enzyme leakage following destruction of hepatocytes [25, 26]. Hepatic necrosis and other liver diseases have been linked to the release of mitochondrial enzymes from the liver [27].

Cytochrome c is a small soluble electron carrier hemeprotein present in large amounts in the inner mitochondrial membrane. It is involved in energy production through transferring electrons from complex III to complex IV. Cytochrome c is released from dying cells either due to apoptosis or necrosis [28, 29].

In the conversion process of herbicide molecules through the oxidation and peroxidation reactions, the cytochrome P450 plays the major role [30]. Herbicide resistance by enhanced detoxification is frequently associated with elevated levels of P450 activity. Enhanced detoxification-based herbicide resistance is particularly difficult to control, because it can involve resistance to multiple, chemically unrelated classes of herbicides [31].

Based on our data, we can argue that NAFLD hepatic cells react to pethoxamid trying detoxifying strategies, ready to undergo cell death to avoid further degeneration. Downregulation of cytochrome can lead to the hypothesis that pethoxamid should not induce herbicide resistance.

The NAFLD model was selected since the accumulation of fat in the liver is only the first step (hit), while oxidative stress resulting in inflammation, stellate cell activation, and fibrogenesis (second hit) progress toward the more critical condition of nonalcoholic steatohepatitis (NASH) [32].

In conclusion, a reanalysis of published studies on biocides toxicity was performed. Missing data on pethoxamid could be partially filled by this preliminary study to identify the liver metabolic fate of NAFLD susceptible cells in the presence of pethoxamid.

## Data Availability

The data used to support the findings of this study are included within the article.
